# Investigating jealous behaviour in dogs

**DOI:** 10.1038/s41598-018-27251-1

**Published:** 2018-06-11

**Authors:** Judit Abdai, Cristina Baño Terencio, Paula Pérez Fraga, Ádám Miklósi

**Affiliations:** 1 Department of Ethology, Eötvös Loránd University, Budapest, H-1117 Hungary; 2 MTA-ELTE Comparative Ethology Research Group, Budapest, H-1117 Hungary

## Abstract

The function of jealous behaviour is to facilitate the maintenance of an important social relationship that is threatened by a third-party, a rival individual. Although jealous behaviour has an important function in gregarious species, it has been investigated almost exclusively in humans. Based on functional similarity between dog-owner and mother-infant attachments, we hypothesised that jealous behaviour can be evoked in dogs, similarly to children. In our study owners focused their attention solely on the test partner, while they ignored their dog. We deployed familiar and unfamiliar dogs as social test partners, and familiar and unfamiliar objects as non-social test partners; all subjects encountered all test partners. Dogs showed more jealous behaviour, i.e. owner-oriented behaviour and attempts to separate the owner and test partner in case of social compared to non-social test partners. Results suggest that jealous behaviour emerges in dogs, and it is functionally similar to that in children observed in similar situations. Alternative explanations like territoriality, dominance rank can be excluded.

## Introduction

Jealous behaviour emerges when an important social relationship with a valued social partner is threatened by a third-party, a rival individual (e.g.^1–3^). Accordingly, an individual displays jealous behaviour if he tries to direct the attention of the valued social partner to itself and attempts to interrupt the interaction between the valued social partner and the social rival in different ways (e.g. pushing, attacking, agonistic displays). Regarding that social relationships can be crucial for survival (e.g. parent-offspring relationship), behaviours that facilitate to maintain these relationships are adaptive. Thus we assume that jealous behaviour emerges in a wide range of animal species despite it has been described almost exclusively in humans (but see^4,5^).

Jealous behaviour is thought to be controlled by a secondary emotion (‘jealousy’) and it is highly debated whether (1) it is present in non-human species and (2) the emotional state underlying this behaviour is comparable to that in humans^4^. It has been assumed that children younger than two years of age do not show jealous behaviour because it requires complex sociocognitive skills^2,6,7^ and they lack the underlying emotional state; however, recent findings suggest that infants from six months of age already display jealous behaviour^8^. Draghi-Lorenz *et al*.^6^ critically reviewed the most important theories about the underlying mental mechanisms that may be required for the appearance of secondary emotions. They conclude that despite the previous views the presence of secondary emotions may not require interpersonal awareness, and that rudimentary forms of these emotions may be present at early development (see also^2^). We suggest that similar theoretical framework as introduced for human infants may apply to non-human species as well. Alternatively, jealousy may not emerge as a distinct emotional state but a blended emotion, i.e. it is the result of an interaction between primary emotions (anger, sadness and may fear)^2,3,9^. This could enable the emergence of jealousy in non-human species, considering that primary emotions are probably present in a wide range of mammalian species^10,11^.

Recent research shows that infants younger than one year display jealous behaviour when the mother focuses her attention to a social test partner, a realistic looking doll in most studies^1,8,12–14^ (but see^15,16^ who used children as social test partners). Across studies researchers have suggested that infants and toddlers display behaviour and facial expressions that may be manifestations of jealousy^1,8,12–14^ (for a review see^2^). These behaviours include closer proximity to, more approach of, increased gaze toward, and more touch of the mother in the presence of a social test partner compared to a non-social test partner. Subjects also displayed more negative affect (angry and sad facial expressions) and lower level of joy. Importantly, subjects showed these behaviours and facial expressions more intensively when the mother was attentive to a social test partner, but not in case of a female stranger^12^. Although in the original studies they referred to all the test partners as *rivals*, we prefer to use the more neutral *test partner* term, as we cannot be sure whether subjects (human infants and toddlers, and non-human species; see below) consider these agents as rivals *per se*.

There have been only a limited number of studies investigating jealous behaviour in non-human species. Observations suggest that jealous behaviour may be present in dogs as well^4,5^. In the questionnaire study by Morris *et al*.^4^ dog owners reported jealous behaviour in social triads, when the owner paid attention to a test partner. Harris & Prouvost^5^ conducted an experimental study with dogs in which they used three test partners: stuffed dog (social test partner); unfamiliar object and book (non-social test partners). Dogs looked longer at the test partner, touched/pushed more often the owner and test partner, and snapped more often the test partner during the stuffed dog condition compared to the non-social test partner conditions. They also found that subjects looked longer at the owner, whined more and tried to get between the owner and test partner more often in the presence of the stuffed dog compared to the book condition, but did not find difference regarding the unfamiliar object. Based on these data it seems that dogs may show jealous behaviour, however, overall dogs did not display a clear distinction between the social and unfamiliar non-social test partners. Thus we caution to interpret these results as an evidence for jealous behaviour in dogs. Although authors claimed that dogs accepted the stuffed dog as real because they sniffed the dog’s anal region and showed agonistic behaviour toward it, we suggest that dogs’ behaviour could be due to distress elicited by the dog-like inanimate object or interest in it. Further, the stuffed dog barked, whined and wagged its tale which are used in communicative interactions; thus even if subjects considered the test partner as a real dog based on its behaviour and physical appearance, these communicative signals used inappropriately might reveal that the test partner is inanimate or make the situation artificial. Also, considering that the tests were carried out in the subjects’ own home, dogs’ behaviour might be considered as territorial aggression.

Several alternative explanations have been raised to account for dogs’ jealous behaviour^4^. Two specific suggestions concern territoriality (see above) and that the behaviour observed in these situations is the result of dog’s rank in hierarchy (see dominance relationships in^17^). One may expect that in multiple-dog households where dogs’ relationship can be described by dominance, the dominant individual may show more intense behaviour when (one of) the subordinate dog(s) engage in exclusive interaction with the owner, that we label as jealous behaviour. Other explanations include protectiveness, playfulness and boredom.

Here we aimed to examine whether dogs show jealous behaviour when the owner gives attention solely to a social test partner. Compared to the previous study^5^ we used real dogs as social test partners (familiar and unfamiliar) and tested dogs at an unfamiliar place (to exclude territorial aggression). We further collected background information about our subjects regarding the households they live in, their jealousy-related behaviour and context in which it occurs, and about their rank in hierarchy in the household. We hypothesised that jealous behaviour (e.g. owner-oriented behaviour (trying to direct the owner’s attention); attempts to separate the owner and test partner) manifests only (mainly) in the presence of social test partners. We further expected that subjects show more test partner-oriented behaviour toward the unfamiliar than the familiar social test partner. We hypothesised that dog’s rank (dominant or subordinate) does not have an effect on their behaviour. Alternatively, the behaviour described as jealous behaviour should be displayed by dogs in the presence of all test partners if it is due to playfulness or boredom; but it should not be displayed in case of the familiar dog if it is due to protectiveness.

Subject dogs encountered four test partners in various order: familiar and unfamiliar dogs as social test partners; and unfamiliar and familiar objects as non-social test partners (conditions named accordingly). The first and last test partner was the familiar dog (a dog from the same household; Familiar dog I and Familiar dog II conditions, respectively), thus overall dogs were observed in five trials. During the test the owner focused his/her attention solely on the test partner while ignoring the subject (see more details in the Methods section). The owner behaved similarly across conditions. We measured dogs’ behaviour (e.g. look, body position) displayed toward the owner, test partner and owner-test partner interaction, and the frequency of attempts to interrupt the owner-test partner interaction.

## Results

All behavioural variables, but the interruption of interaction have been included in the principal component analysis (PCA). Items were grouped into three principal components that accounted for 78.4% of the common variance. The principal components have been labelled as *Interaction-oriented Behaviour* (PC I), *Owner-oriented Behaviour* (PC II) and *Test Partner-oriented Behaviour* (PC III) (Table 1).

**Table 1:** Loadings of items, explained variance, and Eigenvalues of the three factors (PCA). Only loadings greater than 0.5 are shown.

	PC I – Interaction-oriented Behaviour	PC II – Owner-oriented Behaviour	PC III – Test Partner-oriented Behaviour
Body oriented toward the interacting parties	0.860	—	—
Looking at the interaction	0.806	—	—
Staying near the owner	0.685	—	—
Looking at the owner	—	0.885	—
Body oriented toward the owner	—	0.841	—
Body oriented toward the test partner	—	—	0.930
Looking at the test partner	—	—	0.924
Explained variance (%)	32.8	27.1	18.6
Eigenvalues	2.29	1.89	1.30

**Figure 1:**
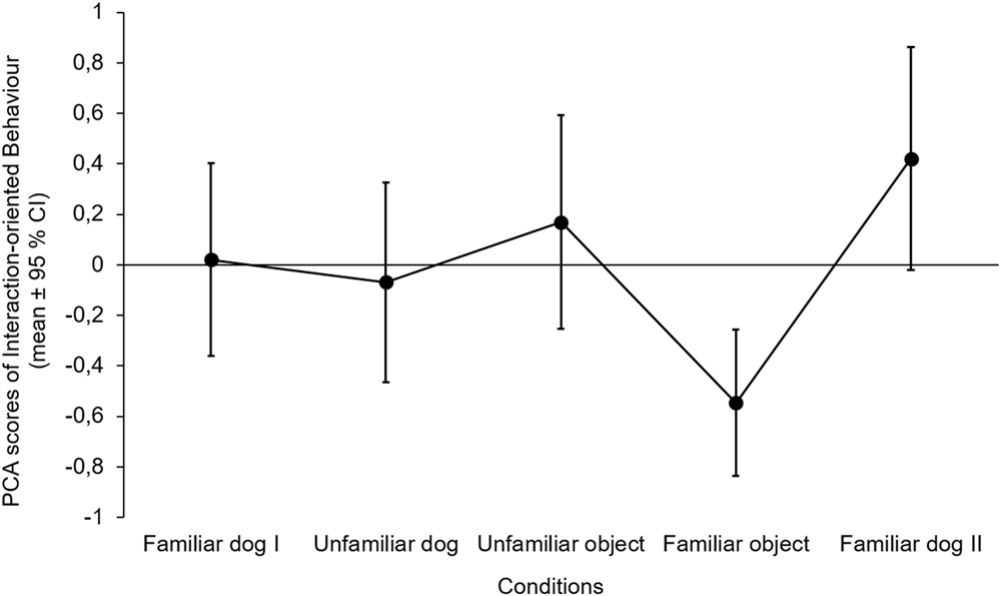
Emergence of *Interaction-oriented Behaviour* in different conditions. Figure shows the original PCA scores before the Box-Cox transformation. The order of Unfamiliar dog, Unfamiliar and Familiar object conditions were counterbalanced among subjects.

There was a significant difference among conditions regarding the *Interaction-oriented Behaviour* (F_4,112_ = 5.053, p = 0.001). Subjects showed less *Interaction-oriented Behaviour* in the Familiar object condition than in case of other test partners, except for the Unfamiliar dog condition (Table 2 and Fig. 1).

**Table 2:** Comparison of the emergence of *Interaction-oriented Behaviour* between conditions (linear GLMM; significant differences are indicated with bold letters).

		Social test partners		Non-social test partners	
		Unfamiliar dog	Familiar dog II	Unfamiliar object	Familiar object
Social test partners	Familiar dog I	p = 0.753	p = 0.576	p = 0.753	**B** ± **SE** = **0**.**457** ± **0**.**168** **t**_**112**_ = **2**.**791** **p** = **0**.**048**
	Unfamiliar dog	—	p = 0.246	p = 0.750	p = 0.246
	Familiar dog II	—	—	p = 0.750	**B** ± **SE** = **0**.**690** ± **0**.**162** **t**_**112**_ = **4**.**267** **p** < **0**.**001**
Non-social test partners	Unfamiliar object	—	—	—	**B** ± **SE** = **0**.**520** ± **0**.**162** **t**_**112**_ = **3**.**213** **p** = **0**.**015**

For significant explanatory variables in the final models, we provide contrast estimates (B ± SE) and t values. Familiar dog I stands for the first, Familiar dog II stands for the last trial.

**Figure 2:**
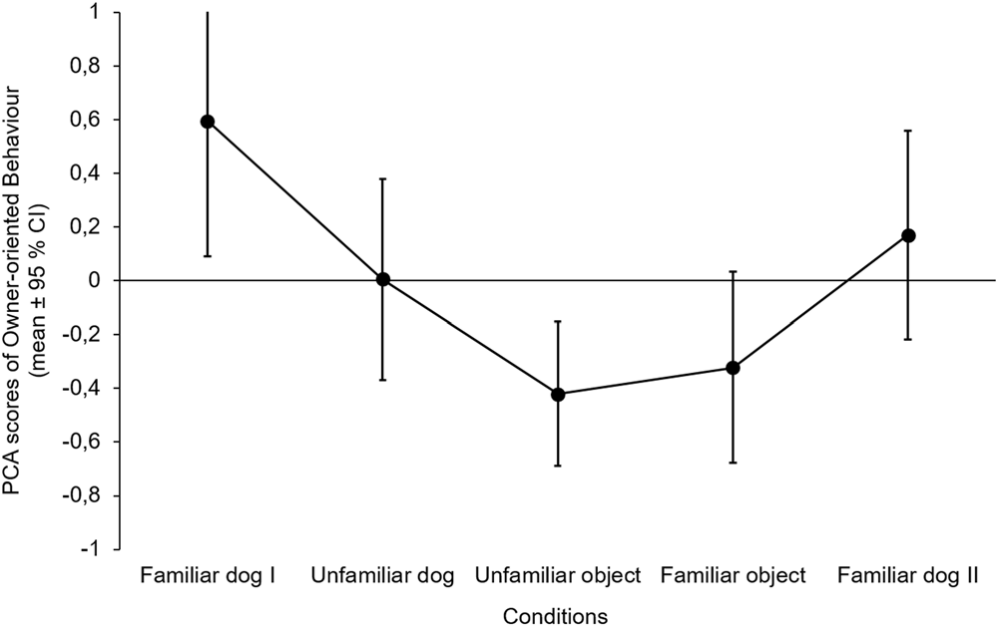
Emergence of *Owner-oriented Behaviour* in different conditions. Figure shows the original PCA scores before Box-Cox transformation. The order of Unfamiliar dog, Unfamiliar and Familiar object conditions were counterbalanced between subjects.

Occurrence of *Owner-oriented Behaviour* also differed between conditions (F_4,112_ = 6.453, p < 0.001). There was no difference between the Familiar dog (I and II) and Unfamiliar dog conditions, but dogs showed more *Owner-oriented Behaviour* in the Familiar dog I condition than in case of the objects, and also in the Familiar dog II condition compared to the Unfamiliar object condition (Table 3 and Fig. 2).

**Table 3:** Comparison of the emergence of *Owner-oriented Behaviour* between conditions (linear GLMM; significant differences are indicated with bold letters).

		Social test partners		Non-social test partners	
		Unfamiliar dog	Familiar dog II	Unfamiliar object	Familiar object
Social test partners	**Familiar dog I**	p = 0.102	p = 0.448	**B** ± **SE** = **0**.**403** ± **0**.**093** **t**_**112**_ = **4**.**356** **p** < **0**.**001**	**B** ± **SE** = **0**.**368** ± **0**.**093** **t**_**112**_ = **3**.**977** **p** = **0**.**001**
	**Unfamiliar dog**	—	p = 0.561	p = 0.286	p = 0.448
	**Familiar dog II**	—	—	**B** ± **SE** = **0**.**265** ± **0**.**091** **t**_**112**_ = **2**.**899** **p** = **0**.**036**	p = 0.090
Non-social test partners	**Unfamiliar object**	—	—	—	p = 0.701

For significant explanatory variables in the final models, we provide contrast estimates (B ± SE) and t values. Familiar dog I stands for the first, Familiar dog II stands for the last trial.

**Figure 3:**
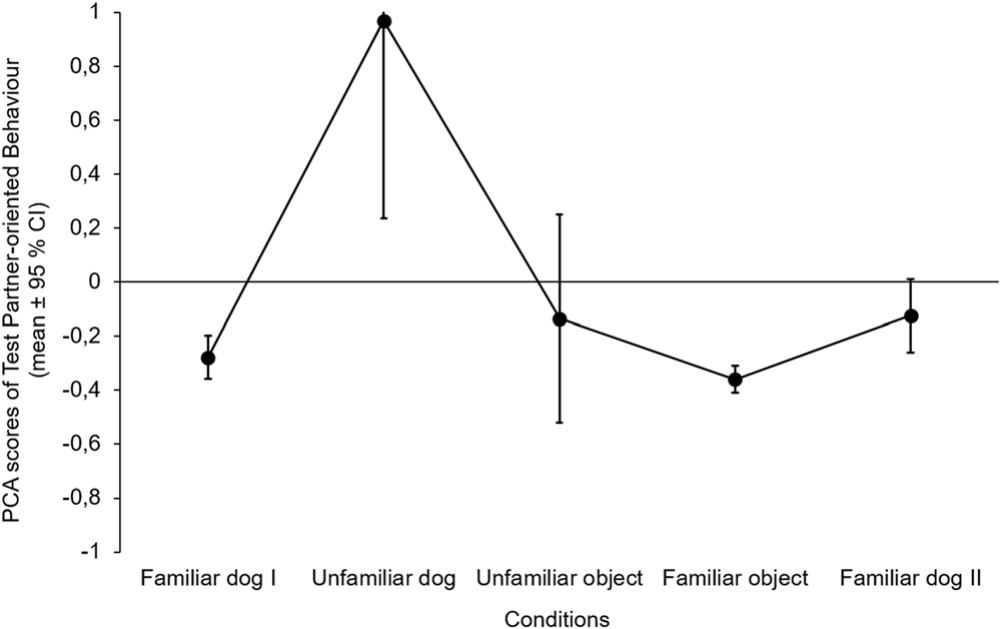
Emergence of *Test Partner-oriented Behaviour* in different conditions. Figure shows the original PCA scores before the Box-Cox transformation. The order of Unfamiliar dog, Unfamiliar and Familiar object conditions were counterbalanced between subjects.

There was a significant difference among conditions in *Test Partner-oriented Behaviour* (F_4,112_ = 9.625, p < 0.001). Subjects showed more *Test Partner-oriented Behaviour* in the Unfamiliar dog condition than in any other conditions; and also showed more behaviour in the Familiar dog II condition compared to the Familiar object condition (Table 4 and Fig. 3).

**Table 4:** Comparison of the emergence of *Test Partner-oriented Behaviour* between conditions (linear GLMM; significant differences are indicated with coloured background).

		Social test partners		Non-social test partners	
		Unfamiliar dog	Familiar dog II	Unfamiliar object	Familiar object
Social test partners	**Familiar dog I**	**B** ± **SE** = **−0**.**562** ± **0**.**128** **t**_**112**_ = **−4**.**381** **p** < **0**.**001**	p = 0.334	p = 0.825	p = 0.539
	**Unfamiliar dog**	—	**B** ± **SE** = **0**.**352** ± **0**.**127** **t**_**112**_ = **−2**.**779** **p** = **0**.**038**	**B** ± **SE** = **0**.**590** ± **0**.**127** **t**_**112**_ = **4**.**652** **p** < **0**.**001**	**B** ± **SE** = **0**.**714** ± **0**.**127** **t**_**112**_ = **5**.**631** **p** < **0**.**001**
	**Familiar dog II**	—	—	p = 0.256	**B** ± **SE** = **0**.**362** ± **0**.**124** **t**_**112**_ = **2**.**924** **p** = **0**.**029**
Non-social test partners	**Unfamiliar object**	—	—	—	p = 0.539

For significant explanatory variables in the final models, we provide contrast estimates (B ± SE) and t values. Familiar dog I stands for the first, Familiar dog II stands for the last trial.

**Figure 4:**
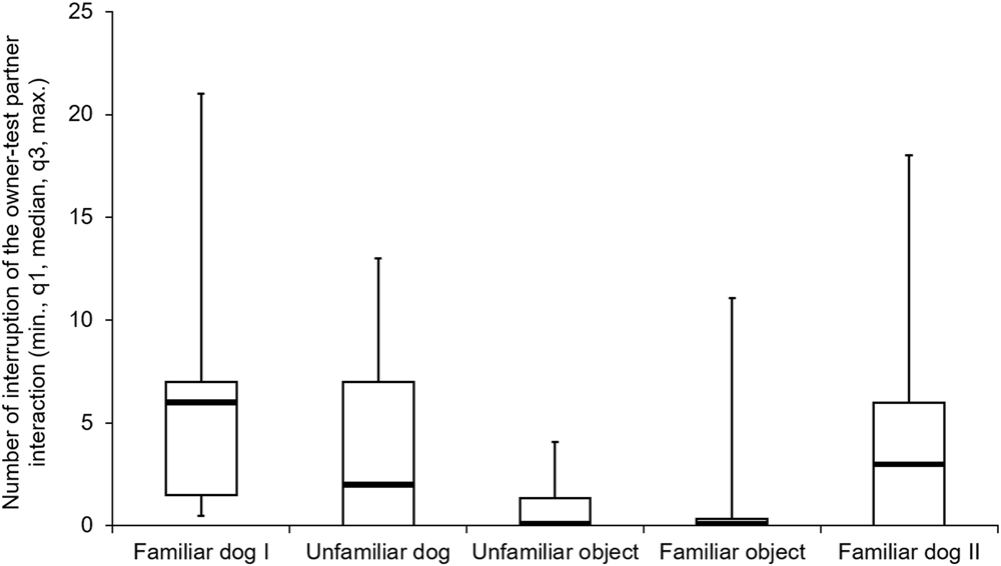
Number of attempts to interrupt the interaction between the owner and test partner in different conditions. The order of Unfamiliar dog, Unfamiliar and Familiar object conditions were counterbalanced between subjects.

Results of the Friedman test show that dogs tried to interrupt the owner-test partner interaction more often in case of social, compared to non-social test partners (N = 22, χ^2^(4) = 30.817, p < 0.001); however, there was no significant difference either within the social test partners or within the non-social test partners (Table 5 and Fig. 4).

**Table 5:** Comparison of attempts to interrupt the owner-test partner interaction between conditions (Friedman test; significant differences are indicated with coloured background).

		Social test partners		Non-social test partners	
		Unfamiliar dog	Familiar dog II	Unfamiliar object	Familiar object
Social test partners	**Familiar dog I**	Z = −0.500 p = 1.000	Z = −0.295 p = 1,000	**Z** = **−1**.**682** **p** = **0**.**004**	**Z** = **−1**.**841** **p** = **0**.**001**
	**Unfamiliar dog**	—	Z = −0.205 p = 1.000	Z = −1.182 p = 0.132	**Z** = **−1**.**341** **p** = **0**.**049**
	**Familiar dog II**	—	—	**Z** = **1**.**386** **p** = **0**.**036**	**Z** = **1**.**545** **p** = **0**.**012**
Non-social test partners	**Unfamiliar object**	—	—	—	Z = −0.159 p = 1.000

Familiar dog I stands for the first, Familiar dog II stands for the last trial.

Trials, order of conditions and dominance rank did not have an effect on *Interaction*-, *Test Partner*- or *Owner-oriented Behaviours* (*Interaction-oriented behaviour*, Trials: F_3,109_ = 1.069, p = 0.365, Condition order: F_5,104_ = 0.567, p = 0.725, Condition x Dominance rank: F_4,73_ = 1.214, p = 0.312, Dominance rank: F_1,77_ = 0.042, p = 0.838; *Test Partner-oriented behaviour*, Trials: F_3,109_ = 1.492, p = 0.221, Condition order: F_5,107_ = 1.262, p = 0.286, Condition × Dominance rank: F_4,73_ = 1.087, p = 0.370, Dominance rank: F_1,77_ = 0.151, p = 0.699; *Owner-oriented behaviour*, Trials: F_3,109_ = 2.110, p = 0.103, Condition order: F_5,104_ = 0.145, p = 0.981, Condition × Dominance rank: F_4,73_ = 0.334, p = 0.854, Dominance rank: F_1,77_ = 0.657, p = 0.420).

## Discussion

Dogs showed more jealous behaviour in case of social compared to non-social test partners, discriminating between the two groups of potential rivals. Considering that dogs showed interest in the owner-unfamiliar object interaction as well, but did not show jealous behaviour (based on our definition; see Introduction), we suggest that the loss of owner’s attention is not enough by itself to elicit the behaviour, but dogs take into account whether the social relationship is threatened by a social agent. Thus it seems that social test partners might be indeed considered as potential rivals from the viewpoint of their relationship with the owner. Regarding that the number of trials, and order of conditions did not have an effect on dogs’ behaviour, and that subjects showed similar behaviour in both familiar dog trials, we can conclude that jealous behaviour is stable over time. Dogs showed functionally similar behaviour as observed in children under two years of age in similar situation that has been referred to as jealousy^1,2,12–14^.

We suggest that test partner-oriented behaviour displayed toward the unfamiliar dog is (at least partially) independent from the jealousy-evoking situation, and dogs were interested in the unfamiliar dog in general. In case of jealousy we would expect agonistic behaviour (e.g. bite attempts, snapping, pushing away the test partner) as test partner-oriented behaviour, because the main function of aggression is to divide resources^18^; however, we did not find evidence on this in the present study (cf.^5^). We suggest that the lack of agonistic behaviour is the result of many owners not allowing their dogs to be aggressive with other dogs in general. The result indicates that the type of social test partner is crucial to study jealous behaviour in dogs (see below).

In the previous experiment conducted with dogs Harris & Prouvost^5^ found longer look at the test partner only in the presence of the social test partner, but looking duration at the owner and number of attempts to interrupt the owner-test partner interaction did not differ between the social and unfamiliar non-social test partners. However, the latter two behaviours are part of the jealous behaviour thus we would expect their emergence only in case of a social test partner, i.e. in the presence of a test partner against which the subject can lose the relationship. In contrast our data show that dogs discriminated between social and non-social test partners regarding their behaviour oriented toward the owner and trying to separate him/her from the test partner. Further, we only found more behaviour oriented toward the test partner in case of the unfamiliar social test partner (see above our argument whether it is part of jealous behaviour). Thus it is possible that in the study by Harris & Prouvost^5^ subjects did not categorize the stuffed dog as an animate or inanimate agent. We propose that the procedure applied in the present study is a better approach to investigate the phenomenon, by using real dogs as social test partners. Considering the difference in dogs’ behaviour toward the familiar and unfamiliar dogs, we further suggest that in future studies familiar dogs may be more suitable to use as social test partners.

As described in the Introduction, territorial aggression and dominance rank within the household has been suggested to explain the behaviour displayed by dogs in such situations (see^4^). Considering that we tested dogs at an unfamiliar place, territorial aggression can be excluded as a causal factor. Similarly, the absence of any association with rank (indicated by the owner) makes it less likely that the displayed behaviour was elicited by dominance aggression. The experimental arrangement makes it unlikely that protectiveness, playfulness and boredom could be major influencing variable. In case of protectiveness we would not expect the behaviour toward the other dog from the household, and in case of playfulness and boredom we would have observed the behaviour in the presence of non-social test partners as well.

Dogs’ behaviour in the present study fulfil the functional description of jealousy; however, it can be argued whether the underlying emotional state shares similarities with the corresponding human emotion. Authors often focus on the emotional state underlying the behaviour, despite it being obscure in human children and adults in contrast to the observable behaviour. We suggest that a behaviour-centred approach may be more fruitful in the future, and this also facilitates comparative investigations.

## Methods

### Ethics

Ethical approval was obtained by the National Animal Experimentation Ethics Committee (PE/EA/3741-4/2016). The experiment was performed in accordance with the EU Directive 2010/63/EU. Owners provided a written consent form to voluntarily permit their dogs to participate in the study.

### Subjects

We tested 25 dogs from multiple-dog households. We could not finish testing one dog because the dog showed distress in the room. Thus we had 24 dogs in the final analysis (11 different breeds and 14 mongrels; 14 females; mean age (year) ± SD 4.9 ± 2.71, see details in the Supplementary Information).

### Questionnaire

Owners filled in an online jealousy questionnaire about the subject dogs prior to the test, the invitation of dogs depended on owners’ report (see responses in the Supplementary Information). The invitation to the test depended on the following questions of the jealousy questionnaire: (1) How jealous do you think your dog is compare to the average dog? (scale from 1 to 10), (2) Who does the dog usually gets jealous of? (3) Where does your dog get jealous? (at home, at unfamiliar places; on a scale from 1 to 5). We only invited dogs the owner of which indicated that the dog shows jealous behaviour toward another dog in the household, and/or other dogs in general. Owners that filled in the questionnaire (overall 631 dogs) gave a mean (±SD) 5.68 (±2.67) jealousy score to their dogs; a mean (±SD) 3.19 (±1.43) score at home, and 2.36 (±1.33) score at unfamiliar places. The tested dogs had a mean (±SD) 7.08 (±1.81) jealousy score given by the owner; a mean (±SD) 4.04 (±1.11) score at home, and 2.65 (±1.34) score at unfamiliar places. In case of one subject the owner filled in the questionnaire for the other dog in the household (i.e. we had information about the familiar dog (test partner), not the subject). Based on the data provided by the owner we could decide whether the subject dog was the dominant or subordinate one in the household (as information about one dog provides information about the other; see below), but we do not have the other information (see Supplementary Information).

In addition we asked the owners four questions to decide whether the dog has a dominant or subordinate rank among dogs in the household (based on^19,20^): (1) *When a stranger comes to the house*, *which dog starts to bark first* (*or if they start to bark together*, *which dog barks more or longer*)?, (2) *Which dog licks more often the other dog’s mouth?*, (3) *If the dogs get food at the same time and at the same spot*, *which dog starts to eat first or eats the other dog’s food?*, and (4) *If the dogs start to fight*, *which dog wins usually?*. We considered the dog dominant if the owner named the subject dog in the answer to the fourth question, or at least twice in the other three questions. We considered the dog as subordinate if the owner indicated another dog from the household in the fourth question, or at least twice in the other three questions. This is a slight change compared to the original criteria used by Pongrácz *et al*.^19,20^ who based the decision on the response to the fourth question, or when the owner uniformly indicated the same dog in response to the other three questions. Pongrácz *et al*.^19,20^ invited dogs based on owners’ response to these questions, thus they could categorise the dogs as dominant or subordinate prior to the test. Considering that in the present study the effect of dominance rank among dogs on their behaviour was not the main question, we did not base the invitation on this. However, we suggest that this change in criteria does not weaken the argument (although these results should be treated with caution).

In case of 7 dogs the owner’s response did not allow for determining whether the dog was a dominant or subordinate individual. In the case of one dog the social status was decided only on the basis of the first three questions because the owner claimed that the subject dog wins in fights only due to the size difference between the dogs (Sheltie vs. Belgian shepherd). In the analysis we had 11 dogs labelled as dominant and 6 labelled as subordinate (see details in the Supplementary Information).

### Test partners

We used four different types of test partners: familiar dog, unfamiliar dog, unfamiliar object and familiar object. The familiar dog was (one of) the other dog(s) from the household (see details in the Supplementary Information). The unfamiliar dog was a middle-sized, neutered female mongrel dog with therapy dog training. Before the test the subject dog and the unfamiliar dog were introduced to each other for about 5 min to see whether any of them shows distress in the presence of the other (in case the owners indicated distress in either the subject or the unfamiliar test partner dog, the introduction was interrupted immediately). We could not test two dogs in the unfamiliar dog condition; however, we tested them in the all other trials. The owner of the unfamiliar dog stood in the room, next to the door she entered during the Unfamiliar dog condition. This allowed her to intervene if needed (e.g. dogs start to fight). She avoided any eye contact with the subject dog and did not talk during the trial.

The unfamiliar object was a remote control car (#32710 RTR Switch Abarth 500, 28 cm × 16 cm × 13 cm) that did not move during the trial. In case of dogs that have already seen the remote control car moving in previous studies (e.g.^21–23^), we used a thermos (25 cm × 14 cm × 14 cm) that had similar colour and size as the car. The familiar object was a newspaper. All subjects encountered all test partners (see above the exception).

### Procedure

Dogs were tested in a 5.2 m × 3 m test room at the Department of Ethology, Eötvös Loránd University. All tests were recorded by four cameras attached to the ceiling.

We had five trials separated by ca. 1–2 min breaks. All trials consisted of two phases: familiarization phase (30 s) and test phase (90 s) that followed each other without a pause. In Trial 1 and 5 the test partner was the familiar dog (condition Familiar dog I and Familiar dog II) to be able to examine the consistency of the behaviour of subjects and the effect of time spent in the room (e.g. fatigue). In Trial 2, 3 and 4 the test partners were the unfamiliar dog, unfamiliar object, and familiar object (conditions named accordingly); we counterbalanced the order of these among subjects.

In Trial 1, the owner and the two dogs entered the room. Dogs could explore the room while the Experimenter (E) informed the owner about the procedure. In the other trials E placed the test partner objects in the room after the owner and subject entered. The subject dog and the unfamiliar dog entered the room at the same time. The familiarization phase started when E left the room. During all familiarization phases the owner ignored both the subject and the test partner; he/she measured the 30 s on a stopwatch.

After the time elapsed, the test phase started during which the owner focused his/her attention on the test partner while continued to ignore the subject. In Trial 1, E told the owner to behave in a way that usually elicits jealousy in the subject dog. Owners mostly choose to pet and talk to the test partner. After Trial 1 ended E told the owners to behave in the same way as with the familiar dog in Trial 1 in the following trials, in order to make the conditions as similar as possible (e.g. in case of the familiar object the owner should read aloud only, if he/she was talking to the familiar dog in Trial 1, and had to repeat at least the most often used words that he/she used before).

### Behavioural and data analyses

Tests were analysed with Solomon Coder 16.06.26. (by András Péter: http://solomoncoder.com). We excluded two dogs from the Unfamiliar dog condition (see above), and we could not code the behaviour of one dog in Trial 1 (Familiar dog I condition) because the owner’s positioning blocked the view of the cameras to the subject.

We measured subjects’ behaviour only in the test phase. Coded behavioural variables were: looking duration at the owner, test partner or owner-test partner interaction (s), duration of body positioned toward the owner, test partner or owner-test partner interaction (s), duration of touching the owner, test partner or owner-test partner interaction (s), duration of moving toward and in parallel with the owner or test partner (owner-, and test partner-related motion) (s), duration of moving toward the owner-test partner interaction (s), and time spent within 0.5 m of the owner. We also coded how many times the subjects tried to interrupt the owner-test partner interaction (move between them). Inter-coder reliability for all variables were tested on a random subsample of the recordings (20% of the subjects) (IBM SPSS 22, Cronbach’s alpha; see results in parenthesis). For the statistical analysis we kept the looking duration (0.749), duration of body position (0.719), time spent next to the owner (0.884), and attempts of interruption data (0.862). However, we excluded the duration of touch (0.649) and motion (0.592) from the analysis due to the low alpha values.

We used IBM SPSS 22 for statistical analyses. Principal component analysis (PCA) with Varimax rotation, Eigenvalue >1 was used for data reduction. We decided the number of factors (three) after the visual inspection of the Scree test. Factor scores were calculated by SPSS automatically, using Regression method.

We used Box-Cox transformation in PC I (Lambda = 0.5), PC II (Lambda = −0.2) and PC III (Lambda = −1.5) as well. We used linear GLMMs to analyse the effect of dominance rank, trial, condition and order of condition on the principal components; the random variable was the ID number assigned to all dogs (within-subject design). Backwards model selection was based on AIC values; the model with the lowest AIC value was kept, we considered a model better when delta AIC was ≥2. For significant explanatory variables in the final models, we provide contrast estimates (B ± SE) and t values.

Frequency of attempts to interrupt the owner-test partner interaction was not normally distributed (Kolmogorov-Smirnov test, Familiar dog I: D_22_ = 0.156, p = 0.172; Unfamiliar dog: D_22_ = 0.249, p = 0.001; Unfamiliar object: D_22_ = 0.340, p < 0.001; Familiar object: D_22_ = 0.400, p < 0.001; Familiar dog II: D_22_ = 0.187, p = 0.044). We used related-samples Friedman test to compare the frequency of interruption of the owner-test partner interaction between conditions. Pairwise comparison by SPSS relied on Dunn’s pairwise post hoc tests followed by Bonferroni correction for multiple testing.

### Data availability

Measurement data of subjects are uploaded as Supplementary Information.

## Electronic supplementary material

Supplementary Information
